# The Relationship Between Illusory Heaviness Sensation and the Motion Speed of Visual Feedback in Gesture-Based Touchless Inputs

**DOI:** 10.3389/fpsyg.2022.811881

**Published:** 2022-05-06

**Authors:** Takahiro Kawabe, Yusuke Ujitoko, Takumi Yokosaka

**Affiliations:** NTT Communication Science Laboratories, Nippon Telegraph and Telephone Corporation, Atsugi, Japan

**Keywords:** illusory heaviness sensation, touchless input system, visual feedback, motion speed, pseudo-haptics

## Abstract

Interaction systems with gesture-based touchless inputs are becoming more common. Nevertheless, perceptual properties of the visual feedback used in the system have not been well documented. We investigated whether the speed of motion shown in visual feedback used in gesture-based touchless inputs could be a cue for the heaviness sensation of an object even when other incidental cues, such as changes in object size and spatial consistencies in direction between gestures and feedback, were eliminated from the stimuli. Participants were asked to make a gesture to grasp and raise/lower disks shown on a horizontal display. The disk’s diameter changed in accordance with the vertical position of the participant’s hand. The results showed that the rate of change in diameter determined the heaviness sensation. When the disks were replaced with concentric gratings having sinusoidal radial intensity and thus the cue of size change was eliminated from the stimuli, the heaviness sensation was dependent on the speed of phase shift (that is, motion) in the grating. It was also found that spatial consistency between the direction of gestures and phase shift was not a critical condition for the heaviness sensation. Finally, the speed of motion served as a critical determinant of the heaviness sensation even when another visual feature (i.e., frame rate) was modulated in a single session, which indicates that the effect of the speed of motion on the heaviness sensation was unlikely due to demanded characteristics. The results indicate that the heaviness sensation for visual feedback of gesture-based touchless inputs is based purely on the speed of the visual feedback motion.

## Introduction

With the development of various sensing technologies, it has become feasible to provide gesture-based touchless inputs to computer systems. Human hand gestures can be detected by using millimeter-wave radar (Lien et al., 2016; Wang et al., 2016a), ultrasonic-wave-based doppler effects (Wang et al., 2016b; Sang et al., 2018), the infrared pyroelectric sensor (Gong et al., 2017), doppler radar (Skaria et al., 2019), depth cameras (Taylor et al., 2016), WIFI (Zhang and Srinivasan, 2016), the combination of electromyography and pressure (McIntosh et al., 2016), and infrared cameras (Leap motion, Ultraleap Inc.).^1^ Based on the detected gesture-based inputs, these systems give sensory feedback to users. Gesture-based touchless operations are becoming more and more practical in many situations, such as digital signage like KIOSK (Georgiou et al., 2019), car systems (Małecki et al., 2020), smart watches (Ogata and Imai, 2015; Xu et al., 2015), and public displays (Muller et al., 2014).

While the use of gesture-based touchless inputs is increasing, only a few aspects have been studied as to how humans perceive the feedback of gesture-based touchless inputs. For example, it is known that users of a touchless input system feel the sense of agency for events which their gestures produce in the external world (Martinez et al., 2017; Evangelou et al., 2021). In particular, the intentional binding phenomenon, which is one of the objective measures for the sense of agency (Haggard et al., 2002), is stronger when the feedback is presented through haptic than visual modalities (Martinez et al., 2017; Evangelou et al., 2021). When users control the virtual object in the display by means of the gesture-based touchless inputs, a three-dimensional change in the appearance of the object serves to give the users a pseudo-haptic impression of the object (Gaucher et al., 2013; Speicher et al., 2019; Kawabe, 2020). Moreover, the virtual button feedback with apparent depth change increases several aspects of evaluative impressions such as attractiveness and efficiency (Speicher et al., 2019). In addition to the virtual button feedback with depth change, other types of feedback from gesture-based input involving position changes (Biocca et al., 2001) and object deformation (Kawabe, 2020) can also produce illusory resistance and stiffness sensations.

In this study, we investigated what image parameters contribute to the generation of an illusory heaviness sensation (i.e., perceived resistance to lifting) when experimental participants manipulated visual objects with gesture-based touchless inputs. Previous studies (Honda et al., 2013; Takamuku and Gomi, 2015; Osumi et al., 2018) have shown that the onset delay of visual feedback for a user’s action caused the heaviness sensation. Moreover, it is also known that the ratio of speed between user’s in-air action and visual feedback motion is a critical parameter in causing the illusory heaviness sensation of visual objects (Jauregui et al., 2014; Taima et al., 2014; Samad et al., 2019). However, in these studies, the effect of image motion speed was not tested separately from the effect of other components, that is, the effect of the change in visual object size and the effect of spatial congruency between gestures and the resulting feedback. Specifically, in these previous studies, the speed change was associated with the change in other types of visual features such as position and size. Moreover, the direction of gesture or action movements was always consistent with the direction of visual feedback (for example, an upward gesture action caused an upward movement of visual feedback), and hence, there was always a spatial consistency between action and visual feedback.

There are two reasons we believe that it is essential to test these components separately. The first reason comes from the scientific point of view. Different brain mechanisms mediate between the perception of motion speed (Mikami et al., 1986) and object size (Tanaka and Fujita, 2015). Hence, to clarify the mechanism underlying the determination of the heaviness sensation, it is meaningful to check whether the heaviness sensation is determined based on image motion speed without accessing object size information. The second reason comes from the previous study (Kawabe, 2020) on the stiffness perception in the touchless input system. The previous study showed that the spatial congruency between user’s action and the feedback did not influence the stiffness perception. In the present study, we tried to check whether the generation of the heaviness sensation in the touchless input system was free from the spatial congruency between the action and its feedback, similar to the stiffness perception.

**Figure 1:**
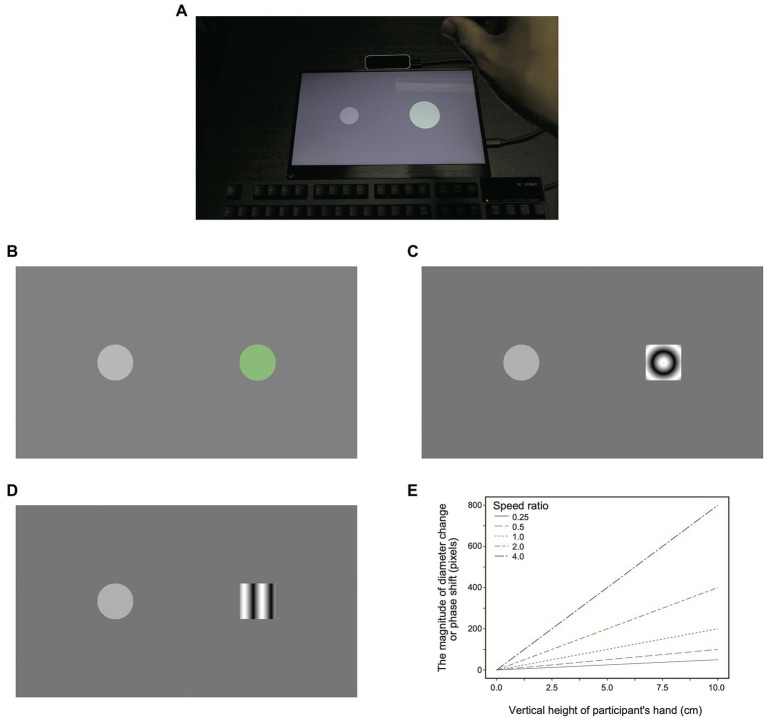
**(A)** A photograph of the scene of the Experiment 1 setting. **(B–D)** Snapshots of typical stimulus display in **(B)** Experiment 1, **(C)** Experiments 2 and 3, and **(D)** Experiment 4. **(E)** A graph plotting the magnitude of diameter change (for Experiment 1) phase shift (for Experiments 2–4) as a function of the vertical height of participant’s hand (cm) in which 0 cm height corresponds to a participant hand’s vertical height at which the stimuli started to change their size and phase.

The purpose of the present study was to examine whether the effect of the ratio of the speed between a user’s action and its visual feedback on the illusory heaviness sensation could still be observed even when both object size changes and the consistency between the direction of the gesture action and the direction of the visual feedback motion were eliminated from the stimuli. We undertook four experiments which are described in more detail below. In each of the experiments, participants stood in front of a horizonal screen which showed images as shown in Figure 1. The participants then moved their right hand to be above an object, they performed a pinching action to simulate grasping the object and moved their hand up and down to simulate lifting and lowering the object. In each case the appearance of the object changed as they brought their hand up and down. In Experiment 1, we sought to show the effect of the ratio of speed between a user’s gesture action and its feedback on illusory heaviness sensation in the situation, where the participants made a gesture to grasp and then raise/lower a visual object, without holding any real object (see Supplementary Video 1; Figures 1A,B). In Experiment 2, by using concentric gratings with sinusoidally varying radial intensity as visual stimuli, we sought to separate the effect of speed from the effect of an object’s size change on the illusory heaviness sensation (see Supplementary Video 2; Figure 1C). In Experiments 3 and 4, we explored how the spatial consistency between the participants’ action and visual feedback influenced the illusory heaviness sensation (see Supplementary Videos 3, 4; Figures 1C,D). The previous experiments controlled only the speed of motion in the feedback within an experimental session. Thus, there was a possibility that the participants judged the heaviness sensation based on the speed of motion as demanded characteristics. In Experiment 5, we confirmed whether the speed of motion in the visual feedback could influence the heaviness sensation even when another visual feature, in addition to the speed of motion, was altered. Work related to the present study was presented elsewhere in an abstract form (Kawabe et al., 2021).

## Experiments 1–4

### Participants

Eleven right-handed people (six females and five males) with the mean age of 25.0 (SD: 7.6) participated in Experiments 1–3. Of these, 10 people (six females and four males) with the mean age of 25.5 (SD: 7.8) participated in Experiment 4. Although the people participated in the experiments in a random order, they were not informed about the specific purpose of the experiments until they completed all of the experiments. The participants reported that they had normal or corrected-to-normal visual acuity. We recruited the participants from outside the laboratory by a hiring agency company in Japan. The participants were paid for their participation. Ethical approval for this study was obtained from the ethics committee at Nippon Telegraph and Telephone Corporation (Approval number: R02-002 by NTT Communication Science Laboratories Ethics Committee). The experiments were conducted according to the principles that have their origin in the Helsinki Declaration. Written informed consent was obtained from all observers in this study.

### Apparatus

As shown in Figure 1A, stimuli were presented on an LCD display (EVC-1301, EVICIV) with the spatial resolution of 1920 × 1,080 pixels (29.2 × 16.5 cm) and a temporal resolution of 60 Hz. A computer (Mac Pro, Apple), on which Windows 10 was installed, controlled the stimulus presentation and data collection. A hand tracker (Leap motion, Ultraleap Inc.) with a temporal resolution of 60 Hz was used to track the hand position of the participants. The generation and presentation of stimuli were controlled by using scripts of Processing 3.^2^ A colorimeter (Bm-5A, Topcon, Japan) was used to measure and linearize the luminance emitted from the display.

### Stimuli

#### Experiment 1

Stimuli consisted of green and gray disks (Figure 1A, Experiment 1) with the CIE coordinates of *x* = 0.31, *y* = 0.38, and *L* = 214.9 cd/m^2^ for the green disk and *x* = 0.31, *y* = 0.33, and *L* = 157.9 cd/m^2^ for the gray disk. When the participant’s right hand was placed above the disk, the color of the disk changed from gray to green. The initial diameter of the disks in both the standard and comparison stimuli was 200 pixels (3.1 cm). We chose the stimulus dimension because we intended to investigate the object’s size that could be manipulated by hand. Two disks were used as the standard and comparison stimuli, which were centered at 400 pixels (6.2 cm) to the left and right of the center of the display. The diameter of the disks changed with the vertical height of the participant’s hand (Figure 1E). As the vertical height increased, the diameter also increased. We controlled the speed ratio for the diameter change, which was defined as the ratio of the speeds of diameter change between the standard and comparison stimuli. In the standard stimulus, the diameter of the disk changed by 20 pixels (0.31 cm) per 1 cm of change in the vertical position of the participant’s hand. In the comparison stimulus, the diameter of the disk changed by 5, 10, 20, 40, and 80 pixels (0.0775, 0.155, 0.31, 0.62, and 1.24 cm) per 1 cm of change in the vertical position of the participant’s hand. Thus, five levels (0.25, 0.5, 1, 2, and 4) of the speed ratios between the standard and test stimuli were employed.

#### Experiments 2 and 3

Stimuli consisted of the gray and green disks as the standard stimuli and a concentric grating as the comparison stimulus. The spatial frequency of the concentric grating was 0.4 cycles per cm. The speed profile of the disk as the standard stimulus was identical to the one as used in Experiment 1. The phase of the grating as the comparison stimulus shifted in accordance with the change in the vertical position of the participant’s hand. Specifically, the phase of the grating shifted by 5, 10, 20, 40, and 80 pixels (0.0775, 0.155, 0.31, 0.62, and 1.24 cm) per 1 cm of change in the vertical position of the participant’s hand. Thus, the speed ratio was again controlled in the five levels (0.25, 0.5, 1, 2, and 4).

#### Experiment 4

Stimuli consisted of a green disk as the standard stimulus and a vertical grating as the comparison stimulus. The standard stimulus was identical to the previous experiments. Instead of the concentric grating as used in Experiment 3, in Experiment 4, we used the vertical grating of which spatial frequency was again 0.4 cycles per cm. Again, five levels of the speed ratio were tested. In each trial, in order to reduce motion aftereffect, the direction of phase shift was randomly determined between leftward and rightward.

#### Common Parameters Among Experiments 1–4

In Experiments 2–4, the comparison stimulus was surrounded by a green outline one pixel wide when the participant’s hand was above the stimuli. In Supplementary Videos 1–4, the disk diameter and grating phase are initially not in motion even when the participant’s hand starts to move; this is because the changes in the disk diameter and grating phase did not occur until the height of the participant’s hand with grasping gestures reached 25 cm from the surface of the display. That is, when the distance between a participant’s hand with grasping gestures and the display surface exceeded 25 cm, the raising/lowering gestures caused the change in size and phase of the stimuli. Figure 1E shows a graph plotting the magnitude of diameter change (for Experiment 1) phase shift (for Experiments 2–4) as a function of the vertical height of participant’s hand (cm) in which 0 cm height corresponds to a participant hand’s vertical height at which the stimuli started to change their size and phase. The minimum distance (25 cm) between the participant’s hand and the display surface was determined so that the participant’s hand would not block the stimuli from the participants’ view. As long as we checked, the time/location of the start/end of the feedback did not influence the heaviness sensation.

### Procedure

#### Experiments 1–4

Each participant was tested individually in a dimly-lit experimental chamber. The participant stood in front of the desk on which the LCD display was put. In each trial, the standard and comparison stimuli were presented on the display, while the positions of the stimuli were randomized between the left and right of the display. The task of the participants was to make a gesture to grasp and raise/lower each stimulus by using their right hand and give a rating for the relative heaviness sensation between the stimuli with a five-point scale (1: the left one much is heavier, 2: the left one is slightly heavier, 3: the two are comparable, 4: the right one is slightly heavier, and 5: the right one is much heavier). They were allowed to repeat their gesture until they felt they could make a satisfactory judgment about the heaviness sensation. In each experiment, each condition was tested four times and thus each observer performed 20 trials, for the five speed ratio conditions and four repetitions. The order of the trials was pseudo-randomized for all the participants.

### Results

#### Experiment 1

The purpose of Experiment 1 was to test whether the heaviness sensation could be induced in a scenario in which the participants manipulated the visual object by making a gesture to grasp and then raise/lower the visual object (i.e., a solid circle, or disk) presented on the display (see Supplementary Video 1; Figures 1A,B). We systematically controlled the amount of change in the diameter of the disk in accordance with the vertical height of the participants’ hand in the raising/lowering gesture (Figure 1E). A larger vertical height of their hand caused the disk with a larger diameter to be displayed. In the experiment, we varied the amount of change in diameter cause by, say, 1 cm increase/decrease in the vertical height of a participant’s hand between standard and comparison stimuli. Here, we defined speed ratio as a ratio of the amount of change in diameter in the standard stimuli to the amount of change in diameter in the comparison stimuli. We hypothesized that consistent with the previous studies (Gaucher et al., 2013; Samad et al., 2019), a lower speed ratio would lead to a heavier sensation for the comparison stimuli. We controlled the ratio of diameter change speeds between standard and comparison disks at five levels [speed ratio (comparison/standard): 0.25, 0.5, 1, 2, and 4]. The positions of the standard and comparison disks were randomized for each trial between the left and right sides of the display. The observers compared the heaviness between the left and right disks on the display and reported the relative magnitude of heaviness sensation between the left and right stimuli on a five-point scale.

**Figure 2:**
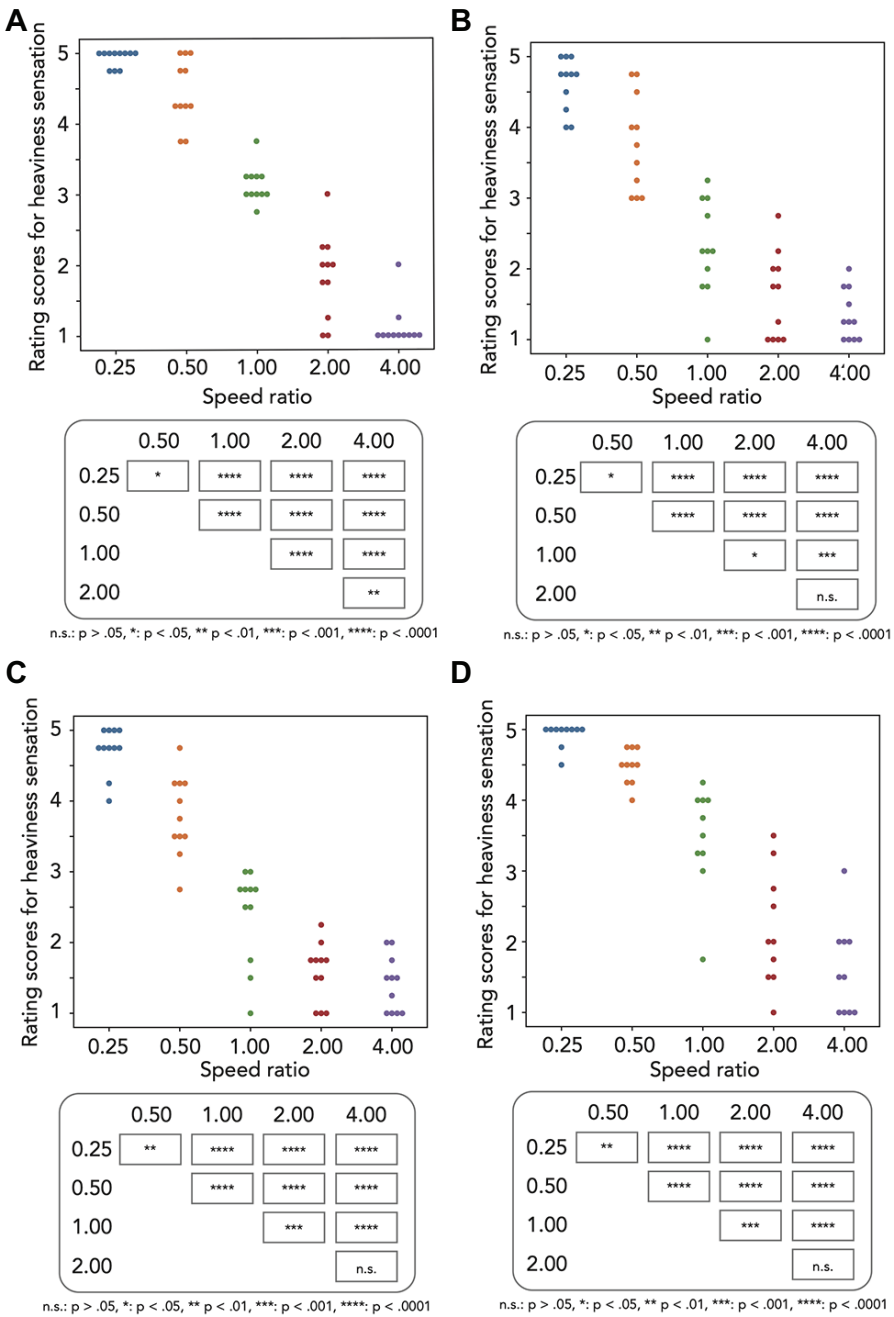
Experimental results. Each graph in the top panel plots the rating scores for heaviness sensation as a function of speed ratio in **(A)** Experiment 1 (*N* = 11), **(B)** Experiment 2 (*N* = 11), **(C)** Experiment 3 (*N* = 11), and **(D)** Experiment 4 (*N* = 10). Each table in the bottom panel indicates the significance level for the multiple comparison based on the significant main effect.

Figure 2A plots individual mean rating scores for heaviness sensation as a function of the speed ratio. In this and the following plots in Figure 2, the rating scores were interpreted as to how the comparison stimulus was rated in comparison with the standard stimulus. Therefore, higher rating scores meant that the comparison stimulus was reported to be heavier than the standard stimulus. Because the rating scores do not in general follow a normal distribution, we first carried out the aligned rank transform (ART; Wobbrock et al., 2011) for the rating scores and next conducted a one-way repeated measures ANOVA with the speed ratio as a within-subject factor. As shown in Table 1, the main effect was significant. Multiple comparison tests with the Bonferroni correction (see the table in the bottom panel of Figure 2A) showed that all pairs of the speed ratio showed significant differences. As a result, the slower change in diameter of the disks caused the heavier sensations of the disk and indicate that consistent with the previous studies (Gaucher et al., 2013; Speicher et al., 2019; Kawabe, 2020), the speed of the appearance change of objects in the display is one of key features for the heaviness sensation.

**Table 1:** ANOVA Table for the results of Experiments 1–4.

Factor	df	df.res	*F*-ratio	*p*-value	*η_P_* ^2^
Experiment 1: Speed ratio	4	40	130.86	<0.0001	0.4816
Experiment 2: Speed ratio	4	40	65.291	<0.0001	0.4644
Experiment 3: Speed ratio	4	40	97.293	<0.0001	0.4755
Experiment 4: Speed ratio	4	36	96.568	<0.0001	0.4777

#### Experiment 2

Because, in the previous experiment, we manipulated the speed ratio by altering the diameter of the disks, the effect of speed change on the heaviness sensation was not separated from any possible effect of the change in the size of disks. To solve this issue, instead of the disks, we used an image of concentric gratings rings with a sinusoidally varying luminance intensity as the comparison stimulus (see Supplementary Video 2; Figure 1C). In this experiment, the increase/decrease in the vertical height of the participant’s hand due to raising/lowering gestures caused an outward (inward) phase motion in the image. By altering the speed of the phase motion, we could test the effect of speed ratio on the heaviness sensation, while keeping the overall stimulus size constant. When the speed ratio was 1, the speed of the diameter change of the disk as the standard stimulus matched the speed of phase shift of the concentric gratings as the comparison stimulus.

Figure 2B plots individual mean rating scores for heaviness sensation as a function of the speed ratio. After the ART, we conducted a one-way repeated measures ANOVA with speed ratio as a within-subject factor. As shown in Table 1, the main effect was significant. Multiple comparison tests (see the table in the bottom panel of Figure 2B) with the Bonferroni correction showed that all pairs but the 2–4 pair of the speed ratio showed significant differences.

The effect of speed on the heaviness sensation was still observed even when the size change cue was eliminated from the comparison stimuli. The results indicate that manipulating the speed of the visual feedback is sufficient to give the heaviness sensation to users for gesture-based touchless system.

One notable difference between Experiment 1 and this experiment was that in Experiment 1, there was a significant difference for the 2–4 pair of the speed ratio while in this experiment, no difference was observed for the pair. When the speed ratio exceeded 1, the rating scores were basically below 3. That is, the comparison stimulus was reported to be lighter than the standard stimulus. The results with no difference for the 2–4 pair of the speed ratio in this experiment indicate that it was more difficult to give the subjective difference in the lightness sensation in this range of the speed ratio by using only the speed cue rather than by using both speed and size. In other words, in order to precisely control the subjective lightness of visual objects in the gesture-based touchless system, it might be better to adjust not only the speed but also the size of the visual object.

#### Experiment 3

In the previous experiments, the motion direction in the stimuli was always consistent with the direction of the participant’s action. That is, when the participants made a gesture to grasp and raise/lower the object on the display, the object expanded/contracted (Experiment 1) or the phase of the grating shifted outward/inward (Experiment 2). On the other hand, if the speed of visual feedback was critical to the manipulation of the heaviness sensation, it was expected that the relationship of the direction between visual motion and participant’s action would not be critical to the determination of the heaviness sensation. To verify the expectation, we changed the comparison stimuli so that the participant’s gesture of raising/lowering the concentric image caused an inward/outward phase shift of the grating (see Supplementary Video 3) as opposed to the outward/inward phase shift used in Experiment 2.

Figure 2C plots individual mean scores for heaviness sensation as a function of the speed ratio. After the ART, we conducted a one-way repeated measures ANOVA with speed ratio as a within-subject factor. As shown in Table 1, the main effect was significant. Multiple comparison tests (see the table in the bottom panel of Figure 2C) with the Bonferroni correction showed that all pairs but the 2–4 pair of the speed ratio showed significant differences.

The pattern of results was similar to the one of Experiment 2. Again, the speed ratio was a strong determinant of the heaviness sensation. Moreover, similar to Experiment 2, no difference in the 2–4 pair was observed. The results indicate that the consistency of the motion direction between visual feedback and participant’s gesture is not a critical condition in causing the heaviness sensation. The speed of visual feedback is effective for the heaviness sensation even when the motion direction between visual feedback and participant’s gesture is reversed.

#### Experiment 4

Although the motion direction in the grating was inconsistent with the direction of participant’s gestures in Experiment 3, the inward motion of the gratings might cause the visual impression of depth change along the axis that was shared with the participant’s gesture, and this shared axis of motion might facilitate perceptual binding between the inward phase motion and participant’s raising/lowering gesture. In this experiment, instead of the concentric grating, we used a vertical grating in which the phase of the grating shifted horizontally in accordance with the participant’s raising/lowering gesture (see Supplementary Video 4; Figure 1D). It was expected that the speed ratio would systematically determine the heaviness sensation even when the participants made a gesture of raising/lowering the vertical grating if a shared motion axis between participant’s gesture and visual objects was not a critical condition.

Figure 2D plots individual mean scores for heaviness sensation as a function of the speed ratio. After the ART, we conducted a one-way repeated measures ANOVA with speed ratio as a within-subject factor. As shown in Table 1, the main effect was significant. Multiple comparison tests (see the table in the bottom panel of Figure 2D) with the Bonferroni correction showed that all pairs but the 2–4 pair of the speed ratio showed significant differences.

Consistent with the previous experiments, the speed ratio affected the pattern of the heaviness sensation. The results indicate that the manipulation of the visual object speed is still effective in giving the heaviness sensation to users who manipulate visual objects *via* gesture-based touchless inputs even when the motion axis is not shared between participant’s gesture and visual objects.

In this experiment, nine out of 10 participants reported mean ratings scores equal to, or greater than, three when the speed ratio was 1.0. The results can be ascribed to the difference in perceived speed between contraction (or expansion) and translational (that is, one-dimensional horizontal) motion. A previous study showed that the contraction and expansion motion was perceived to be faster than rotational and translational motion (Geesaman and Qian, 1998). In this experiment of the present study, the task of the participants was to compare the heaviness sensation between the standard stimulus (expanding/contracting disks) and the comparison stimulus (vertical gratings with translational motion). In this scenario, the comparison stimulus would have been perceived to be slower than the standard stimulus, and this might lead to the heavier sensation for the comparison than the standard stimulus. This interpretation is also consistent with the idea that visual object speed is a determinant of the heaviness sensation in gesture-based touchless systems.

Moreover, a difference in the qualitative appearance of grating motion possibly caused a difference in the results of the experiments. In the stimuli of Experiments 1–3, the phase shift in the concentric grating likely produced the perception of motion-in-depth. On the other hand, the phase shift in the vertical grating produced only horizontal motion without apparent depth change. The difference in the qualitative appearance of motion, in terms of whether stimuli caused the perception of motion-in-depth, might cause the difference of results between a set of Experiments 1–3 and Experiment 4.

## Experiment 5

### Participants

A total of 128 people (64 female and 64 male) with the mean age of 40.19 (SD: 11.13) participated in this experiment. We did not ask the participants’ handedness. We determined the sample size by using MorePower 6.0 (Campbell and Thompson, 2012), with the expectation that our statistical analysis (i.e., a two-way repeated measures of ANOVA with two × five design) involves the power of 0.8 and middle-level effect sizes (*η*^2^ = 0.06). The participants were not informed about the specific purpose of the experiment. An online survey company recruited the participant online. The participants were paid for their participation. Ethical approval for this study was obtained from the ethics committee at Nippon Telegraph and Telephone Corporation (Approval number: R02-009 by NTT Communication Science Laboratories Ethics Committee). The experiments were conducted according to the principles that have their origin in the Helsinki Declaration. Written informed consent was obtained from all observers in this study.

### Apparatus

**Figure 3:**
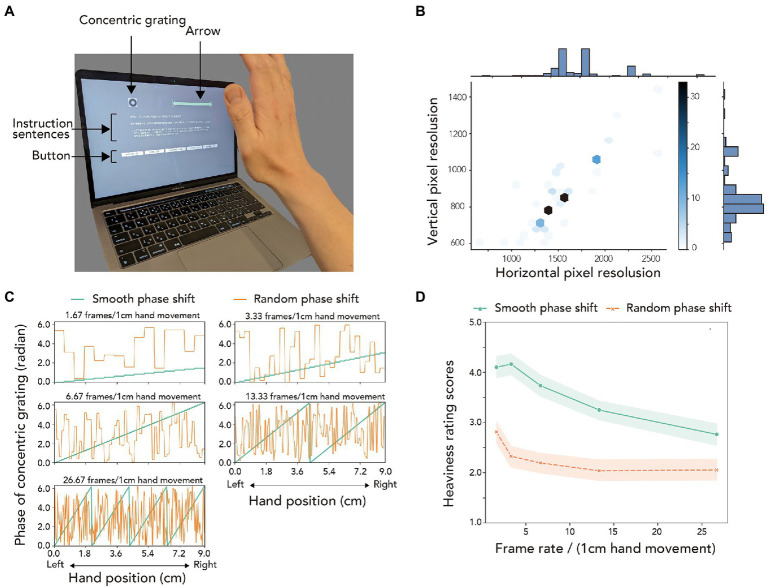
Setup, parameters, and results of Experiment 5. **(A)** A snapshot of the experimental scene of Experiment 5. **(B)** Distribution of horizontal and vertical pixel resolutions of the computer monitors used by the experimental participants to perform the task. The color of the hexagons indicates the number of monitors with the corresponding horizontal and vertical pixel resolutions. **(C)** Variation of the phase of grating with hand position for the smooth and random phase shift conditions. **(D)** Heaviness rating scores for each of the phase shift conditions as a function of the frame rate. Error stripes indicate 95% CI.

The participants performed the task of the experiment by using their own personal computers (Figure 3A; Supplementary Video 5). Figure 3B shows the distribution of the horizontal and vertical pixel resolutions of the computer monitor that the participants used to perform the task. As described in the Procedure section, we tried to specify the relationship between the length of pixels on the monitor and physical length (i.e., cm) by using the method the previous study (Li et al., 2020) introduced. We observed that the pixel length for 1 cm length was 46.766 (SD: 5.488). To track the participants’ right hand position, cameras that were equipped on the computers were used. Mean sampling frequency of the hand tracking by the cameras was 29.76 Hz (SD: 12.27).

### Stimuli

The stimuli again consisted of a concentric grating (see Figure 3A), the phase of which was shifted based on the lateral position of the participant’s hand (see Figure 3C). The concentric grating was presented within a 1.5 cm square area. The spatial frequency of the grating was 1.33 cycles/cm. The luminance contrast of the grating was 0.5. The center of the grating was located 7 cm to the left of the horizontal center of the monitor. In addition, an arrow was displayed on the right side of the monitor. The participant moved their right hand within the spatial extent that this arrow indicated. The length of the arrow was 9 cm. The left side of the arrow was positioned at the horizontal center of the monitor. The color of the arrow was initially white [RGB value (192,192,192)]. While the system detected the participant’s right hand, the color was changed to green [RGB value (255,192,192)]. Stimuli had parameters that were controlled by the following two factors. The first factor was frame rate per 1 cm hand movement. Precisely, the frame rate was controlled in the following five levels (1.67. 3.33, 6.67, 13.33, and 26.67 frames per 1 cm hand movement). The second factor was the phase shift pattern. There were two conditions for the phase shift patterns. As shown in Figure 3B, in a smooth phase shift condition, the phase of the grating was smoothly shifted while in a random phase shift condition, the phase of the grating was shifted in a random fashion. More specifically, in the smooth phase shift condition, the phase of the grating increased with the hand position while in the random phase shift condition, the phase of the grating did not systematically increase with the hand position.

### Procedure

As described in the Apparatus section, because we used the participant’s computer and monitor as apparatuses, the size of the monitor was unknown. Even under such situations, we wanted to control the spatial dimension of stimuli and hence needed to know the relationship between the length of pixels on the monitor and physical length (i.e., cm). To know this, at the initiation of a session, based on Li et al. (2020), our experimental program asked the participants to match the size of a rectangle to the size of a credit card (or other types of a card with the identical spatial dimension to the credit card) which the participants possessed. Based on the reported size of the rectangle, we proportionally controlled the size of stimuli presented in the actual trial so that the stimuli had the spatial dimension that we expected. Following instruction sentences presented on the monitor, the participants set their right hand at a distance of approximately 30 cm from a camera. After detecting the participants’ right hand, the program showed instruction sentences asking the participants to laterally move their right hand in front of the arrow (9 cm in horizontal length) presented in the right upper on the monitor. As shown in Figure 3C, the phase of the concentric grating, which appeared in the left upper on the monitor, was shifted on the basis of the lateral position of the right hand. We recorded the center of the palm in the camera image as the position of the right hand. The participant’s task was to give a rating for the heaviness of the stimuli with a five-point scale (1: very light, 2: moderately light, 3: equivocal, 4: moderately heavy, and 5: very heavy). They reported their rating scores by clicking a button on the monitor or by pressing assigned keys on the keyboard after the participants moved their hand 50% length (i.e., 4.5 cm) of the arrow. Each participant performed 10 trials consisting of two types of phase conditions (smooth or random) and five frame rate conditions (1.67. 3.33, 6.67, 13.33, and 26.6 frames per 1 cm hand movement). The trials were tested with a random order in a single session. Before the formal session, the participants performed a practice session consisting of four trials of two levels of frame rates (the highest and lowest frame rates) × two phase shift patterns (smooth and random phase shifts).

### Results

Although the data in the previous experiments supported the idea that the speed of motion in visual feedback determined the heaviness sensation, there was a concern that the participants in the experiments rated the heaviness sensation without actually feeling “heaviness” on the basis of motion speed because motion speed was a sole parameter that was varied in the experiments. That is, the participants might report the heaviness sensation as task demands.

To address the concern, we conducted an additional experiment wherein other parameter than the speed of motion in visual feedback was also controlled. Specifically, we controlled frame rate, which can be taken as the speed of visual change. For example, in the high and low frame rate conditions, the image of concentric grating was quickly and slowly switched in accordance with the participant’s hand movement, respectively (Figure 3C). At the same time, we manipulated phase shift patterns as the following two conditions. In the “smooth” phase shift condition, the phase of the grating was shifted smoothly as the participant’s hand position changed. On the other hand, in the “random” phase shift condition, the phase of the grating was shifted by a randomly-determined amount. Here, we focused on differences in the speed of motion between the two types of the phase shift conditions. As shown in Figure 3C, in the smooth phase shift condition, the speed of motion in the grating decreases as the frame rate decreases while in the random phase shift condition, mean motion speed (the mean absolute speed of motion speed, precisely) is constantly 0.5*π* in all frame rate condition because the magnitude of phase shift in the random phase shift condition was determined on the basis of uniform distribution ranging from 0 to 2*π* (Here please note that the phase shift with the magnitude larger than *π* causes motion direction opposite to the phase shift with the magnitude smaller than *π*.) We expected that the heaviness rating scores would vary with the frame rate in the smooth phase shift condition, but not in the random phase shift condition, if the participants used motion speed to determine the heaviness. For the null hypothesis, the heaviness rating scores would not vary with the frame rate in both conditions if the dependence of the heaviness rating scores on the speed of motion in visual feedback was due to demanded characteristics.

Figure 3D shows the heaviness rating scores for each of the phase shift conditions as a function of the frame rate. By using the rating scores with the ART, we conducted a two-way repeated measures ANOVA with the phase shift patterns and frame rate as within-participant factors. The results of the ART-ANOVA are shown in Table 2. The main effect of the phase shift pattern was significant. The main effect of the frame rate was also significant. As shown in Supplementary Data 1, multiple comparison tests (Bonferroni procedure) showed that all pairs of the frame rate were significantly deviated from each other. Interaction between the two factors was also significant. The results of the simple main effect analysis and further *post hoc* tests are shown in Supplementary Data 1. In brief, under the smooth phase shift condition, significant differences were found in all pairs of frame rates (*p* < 0.05) except the 0.25–0.5 pair (*p* > 0.05). On the other hand, in the random phase shift condition, significant differences were found between 0.25 and higher frame rates, and between 0.25 and 1 (*p* < 0.05). Effect sizes for the simple main effect of the frame rate were larger in the smooth (*η_P_*^2^ = 0.2847) than the random (*η_P_*^2^ = 0.1083) phase shift conditions.

**Table 2:** ANOVA Table for the results of Experiment 5.

Factor	df	df.res	*F*-ratio	*p*-value	*η_P_* ^2^
<Heaviness rating scores>					
Phase shift patterns	1	127	144.013	<0.0001	0.5314
Frame rate	4	508	54.376	<0.0001	0.2998
Interaction	4	508	17.810	<0.0001	0.1229
<Leftmost hand position>					
Phase shift patterns	1	127	19.7374	<0.0001	0.1345
Frame rate	4	508	1.7446	=0.1388	0.013
Interaction	4	508	1.5945	=0.1744	0.0124
<Mean hand position>					
Phase shift patterns	1	127	0.3072	=0.5804	0.0024
Frame rate	4	508	0.1991	=0.9388	0.0016
Interaction	4	508	1.1768	=0.3201	0.0092
<Rightmost hand position>					
Phase shift patterns	1	127	19.7085	<0.0001	0.1343
Frame rate	4	508	0.7958	=0.5282	0.0062
Interaction	4	508	1.1544	=0.3302	0.0090
<Minimum speed>					
Phase shift patterns	1	127	0.0694	=0.7926	0.0005
Frame rate	4	508	0.3516	=0.8430	0.0028
Interaction	4	508	1.8406	=0.1197	0.0143
<Mean speed>					
Phase shift patterns	1	127	0.5995	=0.4402	0.0047
Frame rate	4	508	0.1576	=0.9595	0.0012
Interaction	4	508	0.4115	=0.8004	0.0032
<Max speed>					
Phase shift patterns	1	127	2.2330	=0.1376	0.0173
Frame rate	4	508	0.9224	=0.4505	0.0072
Interaction	4	508	0.3468	=0.8463	0.0027

The rating scores in the smooth phase shift condition were significantly higher than the scores in the random phase shift condition. The results could be explained in terms of the absolute speed of motion in visual feedback. In the smooth phase shift condition, the speed of motion in the grating ranged between 0.05*π* and 0.88*π*/1 cm hand movement. On the other hand, in the random phase shift condition, the theoretical absolute mean speed of motion in the grating was constantly 0.5*π*. Thus, the results are consistent with the expectation that the heaviness rating scores in the smooth phase shift condition will generally be higher than in the random phase shift condition because the mean speed of motion is lower in the smooth than the random phase shift condition.

We did not ask the participants to gesture to grasp visual objects in this experiment. Nevertheless, we observed the significant effect of the frame rate (that is, the effect of the speed of motion in the smooth phase shift condition) on the heaviness rating scores. The results indicate that the gesture to grasp visual objects is not necessary to cause the heaviness sensation in the touchless input system. The variation of visual stimuli with the participant’s hand position plays a vital role in determining the heaviness rating scores.

Additionally, we analyzed how hand position varied with the two factors we checked. As described above, because we recorded the participant’s right-hand position in the coordinate of the camera image and because the camera’s angle of view depended on the device the participants used, it was not possible, in principle, to directly compute the hand position in the real-world space coordinates. Having said that, within each participant’s environment, the participant likely performed the task with the identical camera’s angle of view and with an almost constant distance between the camera lens and the hand. Therefore, we assumed that we could compare the hand position data among experimental conditions if we standardized it for an individual.

The results of the additional experiment showed that the heaviness rating scores were dependent on the speed of motion in visual feedback even when another visual feature (i.e., the frame rate) than the speed of motion was modulated in an identical session, indicating that the effect of speed of motion on the heaviness sensation is not the product of task demands. Although we expected that the frame rate would not play a role in the random phase shift condition, in the results, the frame rate affected the heaviness judgments. On the other hand, the effect size of the frame rate was larger in the smooth than random phase shift conditions. The results indicate that the speed of motion is possibly a stronger cue to the heaviness sensation than the frame rate.

**Figure 4:**
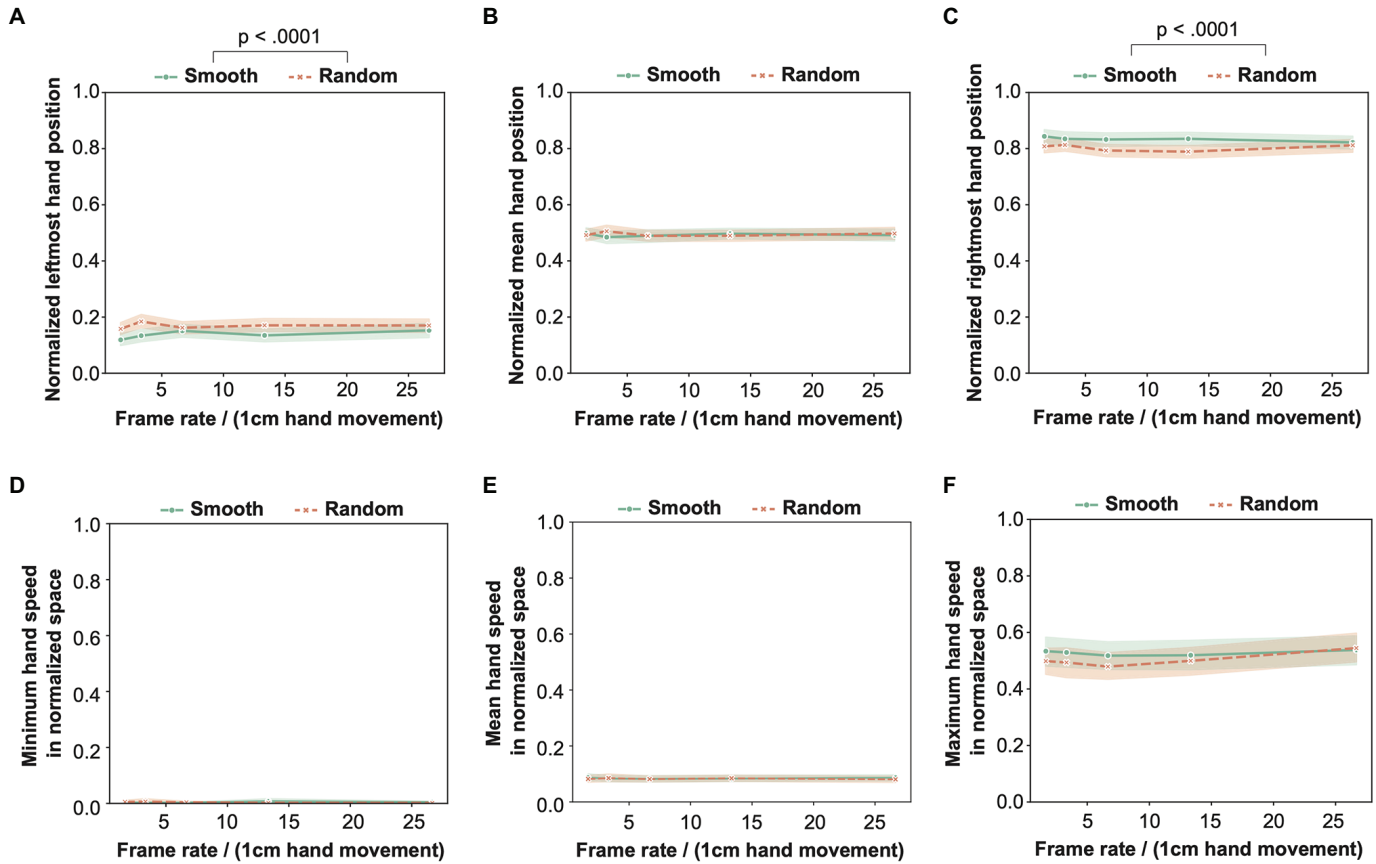
The results of the analysis for the hand position and hand movement speed in normalized hand space. **(A)** Leftmost hand position, **(B)** Mean hand position, **(C)** Rightmost hand position, **(D)** Minimum hand speed, **(E)** Mean hand speed, and **(F)** Maximum hand speed. See text for the detail of these analyses.

Based on the idea, we standardized the hand position for each participant to 0–1, wherein 0 and 1 meant the leftmost and rightmost hand position in individual data. With the normalized data, we individually calculated the leftmost, mean, and rightmost hand positions for each condition and plot their group mean values in Figures 4A–C. Moreover, we calculated the minimum, mean, and maximum speeds of hand movement by dividing the difference in hand position between two samples by a temporal interval between the samples and plot their group mean values in Figures 4D–F. Similar to the analysis with the heaviness rating scores, we conducted the ART-ANOVA for these hand-position data with the phase shift pattern and the frame rate as within-participant factors and show the results of the ART-ANOVA in Table 2. The main effect of the phase shift pattern was significant for the leftmost hand position and the rightmost hand position. Specifically, the leftmost hand position was more biased toward the left in the smooth than the random phase shift condition. Moreover, the rightmost hand position was more biased toward the right in the smooth than the random phase shift condition. The participants moved their right hand with the longer trajectory in the smooth than the random phase shift conditions. The results indicate that phase shift patterns modulate the length of the hand trajectory.

We also checked the relationship between the variation of the heaviness rating scores and the variation of the hand position/motion with the parameters we tested. For the rating scores, the main effect of the frame rate was significant, while for the hand position/motion data, there was no significant main effect of the frame rate. The results indicate that the variation of the hand position/motion is not related to the variation of the heaviness rating scores with the frame rate. On the other hand, the main effect of the phase shift pattern was significant both for the rating scores and the hand position (precisely, the leftmost and rightmost hand positions). The results indicate that the length of the hand trajectory is possibly related to the variation of the rating scores with the phase shift patterns.

## Discussion

To summarize, the present study showed that the speed of visual objects was a critical cue to the heaviness sensation for participants who manipulated visual objects on the display with gesture-based touchless inputs (Experiment 1). The effect of visual object speed on the heaviness sensation was observed even when the size change cue was eliminated from the stimuli (Experiment 2), when the motion direction of the visual object was inconsistent with the one of participant’s gesture (Experiment 3) and when the motion axis was not shared between participant’s gesture and visual objects (Experiment 4). The results indicate that the heaviness sensation with gesture-based touchless inputs is attuned to motion speeds, irrespective of the presence/absence of a size cue and irrespective of the consistencies in motion direction and motion axis between the participant’s gesture and visual object motion. The speed of motion in visual feedback effectively determined the heaviness sensation even when another visual feature (i.e., the frame rate) was modulated in a single session, which indicates that the effect of the speed pf motion on the heaviness sensation unlikely comes from demanded characteristics.

The heaviness sensation based on the speed of visual feedback is possibly relevant to the heaviness sensation due to the delay in visual feedback for an agent’s action. Previous studies (Honda et al., 2013; Takamuku and Gomi, 2015; Osumi et al., 2018) showed that a delay in the visual feedback of an agent’s action caused the illusory heaviness of visual objects or an agent’s limb. When a delay was inserted in the visual feedback of an agent’s action, the average speed of the visual feedback across a certain period of time temporally drops. In view of this, the effect of delay insertion in the visual feedback on heaviness sensations can stem from a similar mechanism for the effect of speed in the visual feedback as shown in this study. Importantly, we used the Leap motion (in Experiments 1–4) and a camera (Experiment 5) to detect the participant’s hand position. These devices, in general, involve processing delay, and thus, there were temporal offsets between the participant’s hand movement and its visual feedback. Given the reported effect of delay on the heaviness sensation in the previous studies, we suggest that the heaviness sensation in our study likely comes from the combination of motion speed in visual feedback and the effect of processing delay.

It was a notable outcome that the consistency of motion direction and motion axis between the participant’s gesture and visual object motion was not critical to the heaviness sensation. The results indicate that an arbitrary combination of user’s gestures and their temporally synchronized visual movements seems to be a sufficient condition to give users the heaviness sensation. By taking this into account, it may be possible to give the heaviness sensation to users for touchless input systems without using more complex computer graphics. That is, it is not always necessary to render the three-dimensional object that can move consistently with users’ gesture patterns. It may be possible to provide the heaviness sensation by adjusting the motion speed of the visual object through image editing, which is computationally more economical than the computation of a three-dimensional object and its movement.

Our results suggest the possibility that various impressions other than heaviness can be given to users through the visual feedback of gesture-based touchless inputs. A previous study (Kawabe, 2020) showed that gesture-based touchless inputs could produce the sensation of object stiffness. In the study, the participants were asked to make a gesture to pull the object on the display both leftward and rightward. Consequently, the participants could feel the various levels of object stiffness depending on the deformation patterns in the object. Since, as shown in the present study, the consistency in direction between the participant’s gesture and visual motion feedback is not critical in generating the heaviness sensation, there is a possibility that the gesture to grasp and raise/lower the visual object can generate the stiffness sensation when the gesture causes the deformation of visual objects in the display. In this way, it may be possible to give various types of sensations to users by appropriately manipulating the visual feedback of gesture-based touchless inputs.

One limitation of the present study is that our results are based on the relative comparison of the heaviness sensation between the standard and comparison stimuli, and thus it is unclear how the heaviness sensation is described in an absolute sense. Future studies need to address how the heaviness sensation caused by speed manipulation is represented, for example, by conducting an experiment wherein a participant compares the illusory heaviness sensation and the strength of force-feedback from external devices. Another limitation is that the heaviness sensation was measured only with a rating method. We believe that our approach is sufficient for the purpose of the present study, which was not to clarify the specific mechanism of the heaviness sensation, but to check whether speed manipulation could alter the subjective magnitude of the heaviness sensation when other cues as described in the Introduction were eliminated from the stimuli. Nevertheless, in order to further pursue the mechanism underlying the heaviness sensation, it will be helpful to use an experimental procedure with a two-alternative forced choice method and calculate, for example, the point of subjective equality and just noticeable differences to understand the bias and sensitivity of the heaviness sensation to the speed ratio.

## Data Availability Statement

The raw data supporting the conclusions of this article will be made available by the authors, without undue reservation.

## Ethics Statement

The studies involving human participants were reviewed and approved by NTT Communication Science Laboratories Ethics Committee. The patients/participants provided their written informed consent to participate in this study.

## Author Contributions

TK, TY, and YU conceived the experiment(s) and reviewed the manuscript. TK conducted the experiment(s) and analyzed the results. All authors contributed to the article and approved the submitted version.

## Funding

This research was conducted as part of the research activities of NTT Laboratories, and therefore no external funds were used.

## Conflict of Interest

TK, TY, and YU are employees of NTT Communication Science Laboratories, which is a basic science research section of Nippon Telegraph and Telecommunication corporation (NTT). There is a pending patent involving the reported research. There are no products in development or marketed products to declare.

## Publisher’s Note

All claims expressed in this article are solely those of the authors and do not necessarily represent those of their affiliated organizations, or those of the publisher, the editors and the reviewers. Any product that may be evaluated in this article, or claim that may be made by its manufacturer, is not guaranteed or endorsed by the publisher.

## Footnotes

^1^ https://www.ultraleap.com/

^2^ https://processing.org

## References

[ref1] Biocca F. Kim J. Choi Y. (2001). Visual touch in virtual environments: an exploratory study of presence, multimodal interfaces, and cross-modal sensory illusions. Presence Teleoperators Virtual Environ. 10, 247–265. doi: 10.1162/105474601300343595

[ref2] Campbell J. I. D. Thompson V. A. (2012). MorePower 6.0 for ANOVA with relational confidence intervals and Bayesian analysis. Behav. Res. Methods 44, 1255–1265. doi: 10.3758/s13428-012-0186-0, PMID: 22437511

[ref3] Evangelou G. Limerick H. Moore J. (2021). I feel it in my fingers! Sense of agency with mid-air haptics. In *2021 IEEE World Haptics Conference (WHC)*; July 6-9, 2021; 727–732.

[ref4] Gaucher P. Argelaguet F. Royan J. Lecuyer A. (2013). A novel 3D carousel based on pseudo-haptic feedback and gestural interaction for virtual showcasing. In *2013 IEEE Symposium on 3D User Interfaces (3DUI)*; March 16–17, 2013; 55–58.

[ref5] Geesaman B. J. Qian N. (1998). The effect of complex motion pattern on speed perception. Vis. Res. 38, 1223–1231. doi: 10.1016/S0042-6989(97)00279-4, PMID: 9666990

[ref6] Georgiou O. Limerick H. Corenthy L. Perry M. Maksymenko M. Frish S.. (2019). Midair haptic interfaces for interactive digital signage and kiosks. In *Extended Abstracts of the 2019 CHI Conference on Human Factors in Computing Systems*; May 4–9, 2019; 1–9.

[ref7] Gong J. Zhang Y. Zhou X. Yang X.-D. (2017). Pyro: thumb-tip gesture recognition using pyroelectric infrared sensing. In *Proceedings of the 30th Annual ACM Symposium on User Interface Software and Technology*; October 22–25, 2017; 553–563.

[ref8] Haggard P. Clark S. Kalogeras J. (2002). Voluntary action and conscious awareness. Nat. Neurosci. 5, 382–385. doi: 10.1038/nn827, PMID: 11896397

[ref9] Honda T. Hagura N. Yoshioka T. Imamizu H. (2013). Imposed visual feedback delay of an action changes mass perception based on the sensory prediction error. Front. Psychol. 4:760. doi: 10.3389/fpsyg.2013.00760, PMID: 24167494 PMC3805955

[ref10] Jauregui D. A. G. Argelaguet F. Olivier A.-H. Marchal M. Multon F. Lecuyer A. (2014). Toward “pseudo-haptic avatars”: modifying the visual animation of self-avatar can simulate the perception of weight lifting. IEEE Trans. Vis. Comput. Graph. 20, 654–661. doi: 10.1109/tvcg.2014.45, PMID: 24650993

[ref11] Kawabe T. (2020). Mid-air action contributes to pseudo-haptic stiffness effects. IEEE Trans. Haptics 13, 18–24. doi: 10.1109/TOH.2019.2961883, PMID: 31880559

[ref12] Kawabe T. Ujitoko Y. Yokosaka T. (2021). Pseudo-heaviness during mid-air gestures is tuned to visual speed. In *2021 IEEE World Haptics Conference (WHC)*; July 6–9, 2021; 580–580.

[ref13] Li Q. Joo S. J. Yeatman J. D. Reinecke K. (2020). Controlling for participants’ viewing distance in large-scale, psychophysical online experiments using a virtual chinrest. Sci. Rep. 10:904. doi: 10.1038/s41598-019-57204-1, PMID: 31969579 PMC6976612

[ref14] Lien J. Gillian N. Karagozler M. E. Amihood P. Schwesig C. Olson E.. (2016). Soli: ubiquitous gesture sensing with millimeter wave radar. ACM Trans. Graph. 35, 1–19. doi: 10.1145/2897824.2925953

[ref15] Małecki K. Nowosielski A. Kowalicki M. (2020). Gesture-based user interface for vehicle on-board system: a questionnaire and research approach. Appl. Sci. 10:6620. doi: 10.3390/app10186620

[ref16] Martinez P. I. C. Pirro S. D. Vi C. T. Subramanian S. (2017). Agency in mid-air interfaces. In *Proceedings of the 2017 CHI Conference on Human Factors in Computing Systems*; May 6–11, 2017; 2426–2439.

[ref17] McIntosh J. McNeill C. Fraser M. Kerber F. Lochtefeld M. Kruger A. (2016). Empress: Practical¨ hand gesture classification with wrist-mounted EMG and pressure sensing. *Proceedings of the 2016 CHI Conference on Human Factors in Computing Systems*; May 7–12, 2016; 2332–2342.

[ref18] Mikami A. Newsome W. T. Wurtz R. H. (1986). Motion selectivity in macaque visual cortex. I. Mechanisms of direction and speed selectivity in extrastriate area MT. J. Neurophysiol. 55, 1308–1327. doi: 10.1152/jn.1986.55.6.1308, PMID: 3016210

[ref19] Muller J. Bailly G. Bossuyt T. Hillgren N. (2014). Mirrortouch: combining touch and mid-air¨ gestures for public displays. In *Proceedings of the 16th International Conference on Human-Computer Interaction With Mobile Devices & Services—MobileHCI ‘14*; September 23–26, 2014; 319–328.

[ref20] Ogata M. Imai M. (2015). Skinwatch. In *Proceedings of the 6th Augmented Human International Conference*; March 9–11, 2015; 21–24.

[ref21] Osumi M. Nobusako S. Zama T. Taniguchi M. Shimada S. Morioka S. (2018). Sensorimotor incongruence alters limb perception and movement. Hum. Mov. Sci. 57, 251–257. doi: 10.1016/j.humov.2017.09.003, PMID: 28943027

[ref22] Samad M. Gatti E. Hermes A. Benko H. Parise C. (2019). Pseudo-haptic weight: changing the perceived weight of virtual objects by manipulating control-display ratio. In *Proceedings of the 2019 CHI Conference on Human Factors in Computing Systems*; May 4–9, 2019; 1–13.

[ref23] Sang Y. Shi L. Liu Y. (2018). Micro hand gesture recognition system using ultrasonic active sensing. IEEE Access 6, 49339–49347. doi: 10.1109/ACCESS.2018.2868268

[ref24] Skaria S. Al-Hourani A. Lech M. Evans R. J. (2019). Hand-gesture recognition using two antenna doppler radar with deep convolutional neural networks. IEEE Sensors J. 19, 3041–3048. doi: 10.1109/JSEN.2019.2892073

[ref25] Speicher M. Ehrlich J. Gentile V. Degraen D. Sorce S. Kruger A. (2019). Pseudo-haptic controls¨ for mid-air finger-based menu interaction. In CHI’19 Extended Abstracts. 1–6.

[ref26] Taima Y. Ban Y. Narumi T. Tanikawa T. Hirose M. (2014). Controlling fatigue while lifting objects using pseudo-haptics in a mixed reality space. In *2014 IEEE Haptics Symposium (HAPTICS)*; February 23–26, 2014; 175–180.

[ref27] Takamuku S. Gomi H. (2015). What you feel is what you see: inverse dynamics estimation underlies the resistive sensation of a delayed cursor. Proc. R. Soc. B Biol. Sci. 282:20150864. doi: 10.1098/rspb.2015.0864, PMID: 26156766 PMC4528553

[ref28] Tanaka S. Fujita I. (2015). Computation of object size in visual cortical area v4 as a neural basis for size constancy. J. Neurosci. 35, 12033–12046. doi: 10.1523/JNEUROSCI.2665-14.2015, PMID: 26311782 PMC6705463

[ref29] Taylor J. Bordeaux L. Cashman T. Corish B. Keskin C. Sharp T.. (2016). Efficient and precise interactive hand tracking through joint, continuous optimization of pose and correspondences. ACM Trans. Graph. 35, 1–12. doi: 10.1145/2897824.2925965

[ref30] Wang W. Liu A. X. Sun K. (2016b). Device-free gesture tracking using acoustic signals. In *Proceedings of the 22nd Annual International Conference on Mobile Computing and Networking*; October 3–7, 2016; 82–94.

[ref31] Wang S. Song J. Lien J. Poupyrev I. Hilliges O. (2016a). Interacting with soli. In *Proceedings of the 29th Annual Symposium on User Interface Software and Technology*; October 16–19, 2016; 851–860.

[ref32] Wobbrock J. O. Findlater L. Gergle D. Higgins J. J. (2011). The aligned rank transform for nonparametric factorial analyses using only ANOVA procedures. In *Proceedings of the 2011 Annual Conference on Human Factors in Computing Systems—CHI’11*; May 7–12, 2011; 143–146.

[ref33] Xu C. Pathak P. H. Mohapatra P. (2015). Finger-writing with smartwatch. In *Proceedings of the 16th International Workshop on Mobile Computing Systems and Applications*; February 12–13, 2015; 9–14.

[ref34] Zhang O. Srinivasan K. (2016). Mudra: User-friendly fine-grained gesture recognition using wifi signals. In *Proceedings of the 12th International on Conference on Emerging Networking Experiments and Technologies*; December 12–15, 2016; 83–96.

