# Supplementary material for: The Relationship Between Illusory Heaviness Sensation and the Motion Speed of Visual Feedback in Gesture-Based Touchless Inputs
Source: Front Psychol. 2022 May 6;13:811881. doi: 10.3389/fpsyg.2022.811881 (PMC9120360; doi:10.3389/fpsyg.2022.811881)
Supplement: Supplementary file 6 [file Data_Sheet_1.PDF]

```
-------------------------------------------------------------
Analysis of Variance of Aligned Rank Transformed Data

Table Type: Repeated Measures Analysis of Variance Table (Type I)
Model: Repeated Measures (aov)
Response: art(Response)
                         Error Df Df.res F value     Pr(>F)    peta
1 Phase_shift            Sb:P_  1    127 144.013 < 2.22e-16 0.53139
***
2 Frame_rate             Sb:F_  4    508  54.376 < 2.22e-16 0.29980
***
3 Phase_shift:Frame_rate S:P_:  4    508  17.810 1.0759e-13 0.12299
***
---
Signif. codes:   0 ‘***’ 0.001 ‘**’ 0.01 ‘*’ 0.05 ‘.’ 0.1 ‘ ’ 1


-------------------------------------------------------------
<Simple main effect of the frame rate when the phase shift is
smooth>

             Error Df Df.res F value     Pr(>F)    peta
1 Frame_rate Sb:F_  4    508  61.143 < 2.22e-16 0.32498 ***
Signif. codes:   0 ‘***’ 0.001 ‘**’ 0.01 ‘*’ 0.05 ‘.’ 0.1 ‘ ’ 1

Multiple comparison tests
 contrast   estimate   SE  df t.ratio p.value
 0.25 - 0.5     -4.0 15.9 508 -0.251  1.0000
 0.25 - 1       64.4 15.9 508  4.040  0.0006
 0.25 - 2      140.8 15.9 508  8.831  <.0001
 0.25 - 4      196.9 15.9 508 12.356  <.0001
 0.5 - 1        68.4 15.9 508  4.291  0.0002
 0.5 - 2       144.7 15.9 508  9.082  <.0001
 0.5 - 4       200.9 15.9 508 12.607  <.0001
 1 - 2          76.4 15.9 508  4.791  <.0001
 1 - 4         132.5 15.9 508  8.316  <.0001
 2 - 4          56.2 15.9 508  3.525  0.0046

-------------------------------------------------------------
<Simple main effect of the frame rate when the phase shift is
random>
Error Df Df.res F value     Pr(>F)    peta
1 Frame_rate Sb:F_  4    508  17.392 2.1965e-13 0.12045 ***
---
Signif. codes:   0 ‘***’ 0.001 ‘**’ 0.01 ‘*’ 0.05 ‘.’ 0.1 ‘ ’ 1

 contrast   estimate   SE  df t.ratio p.value
 0.25 - 0.5    70.57 16.8 508  4.208  0.0003
 0.25 - 1      94.15 16.8 508  5.614  <.0001
 0.25 - 2     121.11 16.8 508  7.221  <.0001
 0.25 - 4     117.52 16.8 508  7.007  <.0001
 0.5 - 1       23.58 16.8 508  1.406  1.0000
 0.5 - 2       50.54 16.8 508  3.013  0.0271
 0.5 - 4       46.95 16.8 508  2.799  0.0532
```

```
 1 - 2         26.96 16.8 508  1.607  1.0000
 1 - 4         23.37 16.8 508  1.393  1.0000
 2 - 4         -3.59 16.8 508 -0.214  1.0000

---------------------------------------------------------------
<Simple main effect of the Phase shift when the phase shift is 0.25>
              Error Df Df.res F value     Pr(>F)    peta
1 Phase_shift Sb:P_  1    127  84.941 8.3135e-16 0.40078 ***
---
Signif. codes:   0 ‘***’ 0.001 ‘**’ 0.01 ‘*’ 0.05 ‘.’ 0.1 ‘ ’ 1

---------------------------------------------------------------
<Simple main effect of the Phase shift when the phase shift is 0.25>

              Error Df Df.res F value     Pr(>F)    peta
1 Phase_shift Sb:P_  1    127  84.941 8.3135e-16 0.40078 ***
---
Signif. codes:   0 ‘***’ 0.001 ‘**’ 0.01 ‘*’ 0.05 ‘.’ 0.1 ‘ ’ 1

---------------------------------------------------------------
<Simple main effect of the Phase shift when the phase shift is 0.5>

              Error Df Df.res F value     Pr(>F)    peta
1 Phase_shift Sb:P_  1    127  143.09 < 2.22e-16 0.52979 ***
---
Signif. codes:   0 ‘***’ 0.001 ‘**’ 0.01 ‘*’ 0.05 ‘.’ 0.1 ‘ ’ 1

---------------------------------------------------------------
<Simple main effect of the Phase shift when the phase shift is 1.0>

              Error Df Df.res F value   Pr(>F)    peta
1 Phase_shift Sb:P_  1    127  86.639 4.98e-16 0.40554 ***
---
Signif. codes:   0 ‘***’ 0.001 ‘**’ 0.01 ‘*’ 0.05 ‘.’ 0.1 ‘ ’ 1

---------------------------------------------------------------
<Simple main effect of the Phase shift when the phase shift is 2.0>

              Error Df Df.res F value     Pr(>F)    peta
1 Phase_shift Sb:P_  1    127  71.144 6.2962e-14 0.35905 ***
---
Signif. codes:   0 ‘***’ 0.001 ‘**’ 0.01 ‘*’ 0.05 ‘.’ 0.1 ‘ ’ 1

---------------------------------------------------------------
<Simple main effect of the Phase shift when the phase shift is 4.0>

              Error Df Df.res F value     Pr(>F)    peta
1 Phase_shift Sb:P_  1    127  37.258 1.1643e-08 0.22683 ***
---
Signif. codes:   0 ‘***’ 0.001 ‘**’ 0.01 ‘*’ 0.05 ‘.’ 0.1 ‘ ’ 1
---------------------------------------------------------------
```
